# Development and Validation of a Model for Postpancreatectomy Hemorrhage Risk

**DOI:** 10.1001/jamanetworkopen.2023.46113

**Published:** 2023-12-06

**Authors:** Emrullah Birgin, Sebastian Hempel, Alina Reeg, Florian Oehme, Annika Schnizer, Johann S. Rink, Matthias F. Froelich, Svetlana Hetjens, Verena Plodeck, Heiner Nebelung, Schaima Abdelhadi, Mohammad Rahbari, Patrick Téoule, Erik Rasbach, Christoph Reissfelder, Jürgen Weitz, Stefan O. Schoenberg, Marius Distler, Nuh N. Rahbari

**Affiliations:** 1 Department of Surgery, Universitätsmedizin Mannheim, Medical Faculty Mannheim, Heidelberg University, Mannheim, Germany; 2 Department of General and Visceral Surgery, Ulm University Hospital, Ulm, Germany; 3 Department of Visceral, Thoracic and Vascular Surgery, Faculty of Medicine and University Hospital Carl Gustav Carus, Technische Universität Dresden, Dresden, Germany; 4 Department of Radiology and Nuclear Medicine, University Medical Centre Mannheim, Heidelberg University, Mannheim, Germany; 5 Department of Medical Statistics and Biomathematics, Medical Faculty Mannheim, Heidelberg University, Mannheim, Germany; 6 Department of Radiology, Faculty of Medicine and University Hospital Carl Gustav Carus, Technische Universität Dresden, Dresden, Germany

## Abstract

**Importance:**

Postpancreatectomy hemorrhage (PPH) due to postoperative pancreatic fistula (POPF) is a life-threatening complication after pancreatoduodenectomy. However, there is no prediction tool for early identification of patients at high risk of late PPH.

**Objective:**

To develop and validate a prediction model for PPH.

**Design, Setting, and Participants:**

This retrospective prognostic study included consecutive patients with clinically relevant POPF who underwent pancreatoduodenectomy from January 1, 2009, to May 20, 2023, at the University Hospital Mannheim (derivation cohort), and from January 1, 2012, to May 31, 2022, at the University Hospital Dresden (validation cohort). Data analysis was performed from May 30 to July 29, 2023.

**Exposure:**

Clinical and radiologic features of PPH.

**Main Outcomes and Measures:**

Accuracy of a predictive risk score of PPH. A multivariate prediction model—the hemorrhage risk score (HRS)—was established in the derivation cohort (n = 139) and validated in the validation cohort (n = 154).

**Results:**

A total of 293 patients (187 [64%] men; median age, 69 [IQR, 60-76] years) were included. The HRS comprised 4 variables with associations: sentinel bleeding (odds ratio [OR], 35.10; 95% CI, 5.58-221.00; *P* < .001), drain fluid culture positive for *Candida* species (OR, 14.40; 95% CI, 2.24-92.20; *P* < .001), and radiologic proof of rim enhancement of (OR, 12.00; 95% CI, 2.08-69.50; *P* = .006) or gas within (OR, 12.10; 95% CI, 2.22-65.50; *P* = .004) a peripancreatic fluid collection. Two risk categories were identified with patients at low risk (0-1 points) and high risk (≥2 points) to develop PPH. Patients with PPH were predicted accurately in the derivation cohort (C index, 0.97) and validation cohort (C index 0.83). The need for more invasive PPH management (74% vs 34%; *P* < .001) and severe complications (49% vs 23%; *P* < .001) were more frequent in high-risk patients compared with low-risk patients.

**Conclusions and Relevance:**

In this retrospective prognostic study, a robust prediction model for PPH was developed and validated. This tool may facilitate early identification of patients at high risk for PPH.

## Key Points

**Question:**

Can perioperative data be used to derive a risk model to predict late postpancreatectomy hemorrhage (PPH) secondary to clinically relevant postoperative pancreatic fistula (cr-POPF) after pancreatoduodenectomy?

**Findings:**

In this prognostic study of 293 patients with cr-POPF after pancreatoduodenectomy, 4 risk factors were identified and incorporated in a risk score model—the hemorrhage risk score—to predict late PPH. The prediction model was well calibrated and capable to predict late PPH with high discrimination values in both the derivation and external validation cohorts.

**Meaning:**

These findings suggest that the hemorrhage risk score can be used as a tool to predict late PPH using perioperative risk factors.

## Teaser

This prognostic study develops a hemorrhage risk score model to predict the development of postpancreatectomy hemorrhage in patients who undergo pancreatoduodenectomy.

## Introduction

Postpancreatectomy hemorrhage (PPH) is the most serious complication after pancreatoduodenectomy.^1^ While the incidence of PPH remains below 5% in high-volume centers, the reported mortality rates in patients with PPH range as high as 21% to 88%.^2^ Therefore, early identification of patients at risk for PPH and their immediate treatment are of clinical importance to reduce perioperative mortality after pancreaticoduodenectomy.

In 2007, the International Study Group of Pancreatic Surgery (ISGPS) defined 2 types of PPH regarding the timing and etiology of PPH. Early PPH is considered to be caused by insufficient hemostasis during surgery and/or coagulopathy, whereas late PPH predominantly results from vascular erosion of peripancreatic vessels secondary to clinically relevant postoperative pancreatic fistula (cr-POPF).^3^ Therefore, much effort has been made to predict the onset of cr-POPF, as it remains a major determinant of morbidity, including PPH as the most severe complication. In 2013, the fistula risk score was introduced^4^ and has since been adopted by many hepatopancreatobiliary centers worldwide in their daily practice.^5,6^ Numerous additional risk models have been introduced to identify patients at risk of developing cr-POPF. However, to our knowledge, no study has specifically addressed the risk of developing late, ie, POPF-associated, PPH. Therefore, the aim of the present study was to address this clinical need and develop and validate a risk prediction tool to identify the subset of patients with cr-POPF at high risk of PPH.

## Methods

### Study Design and Patient Cohort

Consecutive patients who underwent pancreatic surgery between January 1, 2009, and May 20, 2023, at the University Hospital Mannheim (derivation cohort), and between January 1, 2012, and May 31, 2022, at the University Hospital Dresden (validation cohort) were assessed for eligibility. Patients who underwent pancreatoduodenectomy (pylorus-preserving or classic Whipple procedure) for benign and malignant disease were identified. Only patients who developed postoperative cr-POPF as defined by the ISGPS (type B/C) were included for analysis.^7^ Exclusion criteria were arterial resections, pancreaticogastrostomy as the reconstruction method of the pancreatic stump or previous distal pancreatectomy, and patients without postoperative POPF or with postoperative POPF type A. The study was conducted in line with the tenets of the Declaration of Helsinki,^8^ followed the Transparent Reporting of a Multivariable Prediction Model for Individual Prognosis or Diagnosis (TRIPOD) reporting guideline,^9^ and was approved with waiver of informed consent by the University of Heidelberg ethics committee given the study’s retrospective nature.

### Perioperative and Surgical Care

Surgery was performed as an open approach using a Whipple-Kausch or pylorus-preserving pancreatoduodenectomy technique as reported previously.^10,11,12^ Pancreaticojejunostomy was performed in a duct-to-mucosa fashion in a 2-layer technique. Lymphadenectomy was performed in line with the recommendations of the ISGPS.^13^ Nonsuction silicon surgical drains were placed selectively in patients with soft pancreas and/or pancreatic duct diameter less than 5 mm. Amylase and lipase levels were measured from the drain fluid on postoperative days 1 and 3. If the amylase levels in the drain fluid were 3 times higher than the serum level on postoperative day 3, the drains were left in place; otherwise, the drains were removed. All patients who were suspected of having cr-POPF received routine computed tomography to reveal any undrained intra-abdominal fluid collections. If imaging revealed further fluid collection, additional interventional drains were placed by interventional radiologists. Antibiotic treatment with piperacillin and tazobactam in patients with cr-POPF was initiated if signs of abdominal infection or sepsis were present. Antifungal treatment was not part of the initial antimicrobial therapy. Before initiation of antimicrobial therapy, cultures were obtained from abdominal drain fluid (operative and/or percutaneous drains). Patients with sentinel bleeding immediately received a triphasic computed tomography angiography and were transferred to the intermediate care unit.^14^

### Definitions and Data Acquisition

We extracted clinicopathologic and postoperative data from prospectively maintained databases.^10,11,15^ Postoperative outcomes included any type of complication according to the Clavien-Dindo classification within 90 days of surgery, specific postpancreatectomy complications according to the ISGPS definitions, and 90-day mortality rate.^3,7,16,17,18,19^ The day of surgery was defined as postoperative day 0, whereas the day after surgery was defined as postoperative day 1. Occurrence of sentinel bleeding, defined as minor blood loss via surgical drains or the gastrointestinal tract before a major bleeding event, was included in the analyses as a clinical feature.^14,20^

### Radiologic Assessment

Two experienced radiologists at each institution (F.O., J.S.R., M.F.F., and V.P.) were blinded to the patients’ outcomes and assessed the postoperative computed tomography imaging findings that are known to be associated with late-onset PPH.^21,22,23^ These findings include the presence of a peripancreatic fluid collection with gas and rim enhancement of peripancreatic intra-abdominal fluid collection (eFigure 1 in Supplement 1).

### Sample Size Calculation

The incidence of late PPH was anticipated to be 10% in the derivation cohort and the number of candidate predictors to be included in the multivariate model was restricted to 4 variables. Assuming an estimated input C statistic of 0.95, a shrinkage factor of 0.9, and an optimism of 0.05 in the apparent *R*^2^ value, a minimum sample size of 139 patients was calculated in line with recommendations to develop a clinical prediction model.^24^

### Statistical Analysis

Data analysis was performed from May 30 to July 29, 2023. The statistical analysis was performed using R, version 4.1.2 (R Foundation for Statistical Computing). Categorical parameters are expressed as frequencies and were compared using the Pearson χ^2^ test or Fisher exact test. Continuous variables are reported as mean (SD) or median (IQR), depending on the distribution pattern, and were compared using the 2-tailed *t* test or Mann-Whitney test. In the derivation cohort, a multivariate regression analysis for significant variables with *P* < .05 on univariate analysis was performed. Variables that achieved *P* < .05 were assigned scores proportional to the β regression coefficient values divided by 2 to the nearest integer. The Youden index was used to determine a threshold value for a high risk of PPH. Three risk categories were defined based on the determined risk scores including low and high risk of PPH. The Cochran-Armitage trend test was used to evaluate the risk for PPH among the risk groups. The derived predictive model was internally validated by 1000 bootstrap samples and externally validated in the validation cohort.^25^ The concordance index (C index) was used to assess the model performance. The predictive strength and model accuracy was measured by the Nagelkerke *R*^2^ and the Brier scores.^25^ A calibration plot was generated to visualize the agreement between predicted and observed outcomes.^9^ A receiver operating characteristic curve analysis with its corresponding area under the curve (AUC) was used to assess the predictive value of the developed hemorrhage risk score model to predict PPH. *P* values <.05 were defined as statistically significant.

## Results

### Study Population

**Figure 1.**
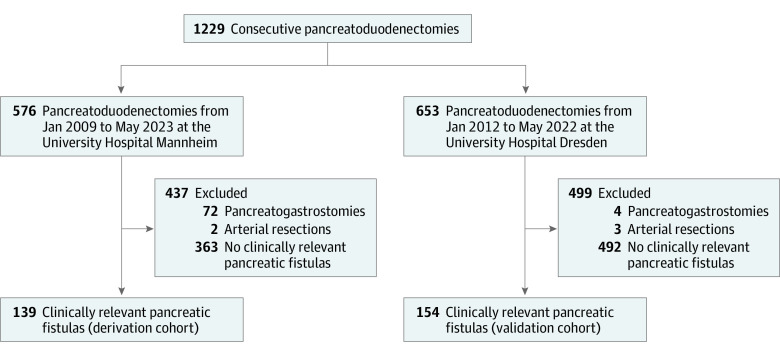
Patient Flowchart

A total of 1229 consecutive patients with pancreatoduodenectomies were screened for eligibility, of whom 293 individuals (187 [64%] men; 106 [36%] women) met the inclusion criteria and were included, with 139 patients in the derivation cohort and 154 in the validation cohort (Figure 1). The median age of the patients was 69 (IQR, 60-76) years, and 74 patients developed late PPH after a median of 13 (IQR, 9-18) days. The demographic characteristics of the derivation and validation cohorts are detailed in eTable 1 in Supplement 1. There was a significantly higher frequency of patients with cardiovascular diseases (46% vs 18%; *P* < .001) and use of antithrombotic drugs (30% vs 5%; *P* < .001) in the derivation cohort compared with the validation cohort, while the baseline and operative characteristics and postoperative outcomes were well balanced between the study cohorts.

### Risk Factors for PPH

To scrutinize risk factors for PPH, we performed logistic regression analysis in the derivation cohort (eTable 2 in Supplement 1). We considered 35 variables (19 preoperative, 3 intraoperative, and 13 postoperative) with known predictive value for PPH.^26,27,28,29,30,31^ Univariate analysis of these risk factors found that postoperative sentinel bleeding (odds ratio [OR], 35.0; 95% CI, 2.65-21.20; *P* < .001), rim enhancement (OR, 10.4; 95% CI, 3.7-29.7; *P* < .001) of or gas within a peripancreatic fluid collection (OR, 18.7; 95% CI, 5.7-61.5; *P* < .001), and distinct drain microbiota pattern with predominance of *Candida* species (OR, 7.5; 95% CI, 2.7-21.2; *P* < .001) were associated with PPH. On multivariate analysis, all 4 variables were confirmed as independent predictors of late PPH: sentinel bleeding (OR, 35.10; 95% CI, 5.58-221.00; *P* < .001), *Candida* species drainage (OR, 14.40; 95% CI, 2.24-92.20; *P* < .001), fluid collection with gas (OR, 12.10; 95% CI, 2.22-65.50; *P* = .004), and rim enhancement (OR, 12.00; 95% CI, 2.08-69.50; *P* = .006) (Table 1). A total of 42 patients had at least 1 risk factor (30%) and 15 patients had at least 2 risk factors (11%). The most common risk factor was fluid collection with gas (37 [27%]), while sentinel bleeding (19 [14%]) was a relatively rare event.

**Table 1.** Multivariate Regression Analysis of Variables Associated With Postpancreatectomy Hemorrhage in the Derivation Cohort

Model and variable	OR (95% CI)	*P* value	β Regression coefficient	Points^a^
Clinical features				
Sentinel bleeding	35.10 (5.58-221.00)	<.001	3.56	2
Positive drainage culture				
*Candida* species	14.40 (2.24-92.20)	<.001	2.67	1
Radiologic features				
Fluid collection with gas	12.10 (2.22-65.50)	.004	2.49	1
Rim enhancement	12.00 (2.08-69.50)	.006	2.49	1

Abbreviation: OR, odds ratio.

^a^ The scoring points in the final model were assigned to the β regression coefficient values divided by 2 to the nearest integer. Patients who meet all criteria have 5 points and patients lacking all criteria have 0 points. A high risk of postpancreatectomy hemorrhage is present in patients having 2 points and higher.

### Hemorrhage Risk Score

**Figure 2.**
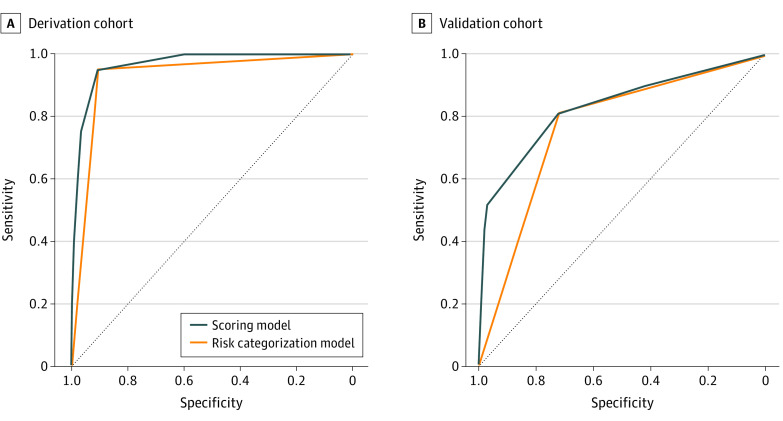
Receiver Operating Characteristic Curves in the Derivation and Validation Cohorts A, The receiver operating characteristic curve analysis of the hemorrhage risk score to predict postpancreatectomy hemorrhage in the derivation cohort is displayed. An area under the curve (AUC) of 0.97 (95% CI, 0.94-0.99) was calculated for the scoring model from 0 to 4 points. The AUC of the risk categorization model (low vs high risk) yielded a value of 0.93 (95% CI, 0.87-0.98). B, In the validation cohort, an AUC of 0.83 (95% CI, 0.76-0.90) was calculated for the scoring model, and an AUC of 0.77 (95% CI, 0.70-0.84) for the risk categorization model (low vs high risk).

In the next step, the hemorrhage risk score (HRS) model was established based on the β regression coefficients of the 4 identified independent risk factors. Two points were allocated to the risk factor sentinel bleeding, while the other 3 risk factors were each allocated a single point (Table 1). The ability to discriminate between PPH and no PPH was analyzed by the AUC and found an AUC of 0.97 (95% CI, 0.94-0.99) (Figure 2A). All 20 patients with PPH had at least 1 point in the model, and PPH was predicted correctly by the HRS. To measure the accuracy of the HRS, an internal validation was subsequently performed using the bootstrap resampling method with 1000 samples. The discrimination ability of the internal validation model was high (C index, 0.93). The calibration plot is displayed in eFigure 2A in Supplement 1 and indicated good calibration between the predicted and observed PPH frequency. The goodness-of-fit test for the HRS showed a correlation between the predictors and PPH, with a Nagelkerke *R*^2^ value of 0.70. The accuracy of the HRS was verified by the Brier score and yielded a high prediction value of 0.05. An increasing trend of PPH was found in patients having higher scores in the HRS (0 points, n = 0; 1 point, n = 1; 2 points, n = 4; 3 points, n = 7; 4 points, n = 5; and 5 points, n = 3; *P* < .001).

### Risk Categorization

Next, we assessed the incremental outcomes of risk factors in the HRS. Postpancreatectomy hemorrhage rates were detected in patients having 0, 1 (3%), 2 (36%), 3 (70%), 4 (83%), and 5 (100%) points in the HRS. A cutoff value of 2 points was determined as a clinically relevant threshold for a high PPH risk with a sensitivity of 95.0% and specificity of 90.8% (AUC, 0.97; 95% CI, 0.94-0.99). Therefore, we categorized the total score in risk categories as low risk (0-1 points) and high risk (≥2 points) (Figure 2A). The PPH rates were 1% in the low-risk category and 63% in the high-risk category (eTable 3 in Supplement 1). The risk categorization model was recalibrated and internally validated with bootstrap resampling. The discrimination of this model was high (C index, 0.857) and accurate (Brier score, 0.060), and again yielded excellent calibration values as indicated by the calibration plot (eFigure 2 in Supplement 1) and Nagelkerke *R*^2^ value of 0.654.

### Validation of the HRS

**Figure 3.**
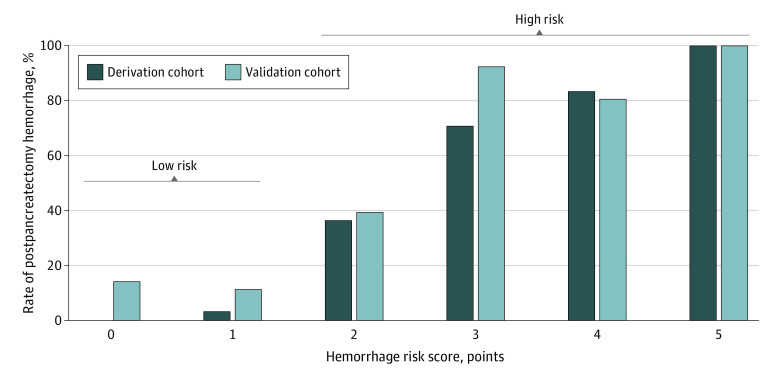
Postpancreatectomy Hemorrhage Rate in the Derivation and Validation Cohorts With Regard to the Postpancreatectomy Hemorrhage Risk Score and Risk Classification

External validation of our findings revealed PPH rates in patients having 0 (14%), 1 (11%), 2 (39%), 3 (92%), 4 (80%), and 5 (100%) points in the HRS (Figure 3). Regarding the risk categories, PPH rates of 12% were observed in low-risk category and 61% in the high-risk category (eTable 3 in Supplement 1). The C index of the HRS was 0.83 (95% CI, 0.76-0.90) and of the risk categorization model was 0.77 (95% CI, 0.69-0.84) in predicting PPH in the validation cohort (Figure 2B). An increasing trend of PPH risk was confirmed from 0 to 5 points and from low to high-risk groups. The HRS predicted 44 of 54 patients with observed PPH, while only 10 of 82 patients having 0 to 1 point in the HRS developed PPH and were not identified by the HRS.

### Association of HRS With Postoperative Outcomes

To further investigate the overall postoperative outcomes of the HRS, the total cohort (derivation and validation) was stratified by HRS risk categories (Table 2). Patients at high risk were more likely to develop severe postoperative complications, which required more invasive interventions, surgical revisions, and completion pancreatectomies compared with patients at low risk. In addition, the risk categories were not only associated with an increasing overall incidence of PPH, but also with a higher mortality rate in patients at high risk compared with those at low risk.

**Table 2.** Postoperative Outcomes and Risk Stratification Based on the Hemorrhage Risk Score in the Total Cohort

Variable	No. (%)		*P* value
	Low risk (0-1 points) (n=191)	High risk (≥2 points) (n=102)	
Postoperative complications^a^			
Grade I-IIIa	126 (66)	30 (29)	<.001
Grade IIIb-IVb	44 (23)	50 (49)	
Interventional treatment	64 (34)	74 (73)	<.001
Surgical revision	55 (29)	62 (61)	<.001
Completion pancreatectomy	28 (15)	32 (31)	.001
PPH			
Type B	3 (2)	20 (20)	<.001
Type C	8 (4)	43 (42)	
90-d Mortality rate	21 (11)	22 (22)	<.001

Abbreviation: PPH, postpancreatectomy hemorrhage.

^a^ In line with the Clavien-Dindo classification.

## Discussion

Because pancreatoduodenectomy carries a high risk for postoperative morbidity, risk stratification has been a major scope of research over the past decades. However, early prediction and management of PPH resulting from POPF-associated vascular erosion remains a clinical challenge. To our knowledge, there has been no prediction tool available to assess the risk of PPH in the subset of patients with cr-POPF. In this study, we assessed 35 different candidate predictors of PPH and identified 4 independent risk factors for PPH. These 4 variables were compiled to a risk model to predict PPH in patients with cr-POPF. The HRS effectively stratified patients at high risk for PPH with a C index statistic of 0.93 in the derivation cohort and 0.77 in the validation cohort. Most notably, we detected more adverse postoperative outcomes and invasive PPH treatments in patients with high-risk profiles based on the HRS.

Of the 4 variables in the HRS, most likely sentinel bleeding has been so far considered a major clinical predictor for PPH. Sentinel bleeding precedes major hemorrhage in patients with cr-POPF after pancreatoduodenectomy with PPH rates of 30% to 100%.^32,33^ This study supports the high predictive value of sentinel bleeding in predicting PPH. Moreover, our data identified 2 distinct radiologic features: rim enhancement of or gas within a peripancreatic fluid collection, as well as a characteristic microbiota signature with a predominance of *Candida* species to be independently associated with PPH. Taken together, our findings provide evidence that PPH in patients with cr-POPF emerges from a *Candida* species–triggered abscess in the vicinity of the pancreaticojejunostomy with subsequent erosion of peripancreatic vessels. In small patient cohorts, 2 studies suggested a correlation between *Candida* species and cr-POPF (n = 21) as well as between *Candida* species and PPH (n = 24).^34,35^ Despite *Candida* species being part of the physiologic intestinal microbiome, a disbalance of *Candida* species to other microorganisms is believed to cause infections with subsequent high mortality rates ranging between 28% and 88%, comparable with systemic candidiasis.^36^ Intra-abdominal candidiasis typically presents with bacterial coinfections (ie, *Escherichia coli*) manifesting as intra-abdominal abscess and distinct radiologic features.^37^ However, our study found that *Candida* species was independently associated with PPH in the multivariate analysis, which raises the question of prophylactic antifungal treatment. Although the prognosis of patients with occult *Candida* infection after gastrointestinal surgery can be improved by prophylactic treatment with fluconazole, to our knowledge, there is currently no role for antifungal therapy in the early postoperative setting after pancreatoduodenectomy.^38,39^

There is further limited evidence for strong radiologic features predicting PPH, with fluid collection around the pancreatic anastomosis and signs of abdominal fluid infection, such as rim enhancement of or gas within a peripancreatic fluid collection, found to be associated with PPH.^26,27,28^ Thus, we advocate draining new peripancreatic fluid collections to prevent subsequent PPH. Another study found peripancreatic fluid collection (OR, 3.8; 95% CI, 1.0-14.3), perianastomotic air bubbles (OR, 3.9; 95% CI, 1.0-14.6), and a defect of the pancreaticojejunostomy (OR, 21.2; 95% CI, 5.7-79.2) shown on computed tomography as independent predictors of PPH in a cohort of 166 patients with cr-POPF.^40^ Nevertheless, we believe that a defect of the pancreaticojejunostomy might not be easily detected or differentiated from peripancreatic fluid collections on imaging, whereas perianastomotic air bubbles or fluid collections are unambiguous findings.

To our knowledge, there are no other risk models available to predict PPH in patients undergoing pancreatoduodenectomy. Other studies assessed the preoperative and intraoperative factors observed with the onset of PPH and reported male sex, body mass index greater than or equal to 25 (calculated as weight in kilograms divided by height in meters squared), and the absence of diabetes as independent risk factors for PPH but did not identify any intraoperative risk factors associated with PPH.^27,29^ In the present study, none of the preoperative or intraoperative factors was associated with PPH. This finding was potentially due to our homogeneous cohort of patients with cr-POPF. In general, the use of preoperative factors for risk stratification remains of minor clinical value as pancreatoduodenectomy would potentially not be withheld from patients with a surgical indication. The HRS may be a useful tool to predict PPH, the importance of which is further highlighted by the potentially lethal outcome for patients with PPH after pancreatoduodenectomy. Our model is applicable in clinical practice because it is easy to use, fast, and safe to perform since all patients with cr-POPF usually undergo at least 1 computed tomography scan and routine microbial testing of the drain fluid in the postoperative course. Compared with other common risk models used in pancreatic surgery, namely, the fistula risk score, the HRS reached comparably high discrimination values in the derivation cohorts (C index statistic >0.9). Notwithstanding, the fistula risk score did not reach equivalent C-statistic values in other validation cohorts, with values ranging from 0.6 to 0.8; therefore, the HRS needs to be further tested in multi-institutional cohorts to elucidate its final discrimination ability.^41^

### Limitations

The present study has limitations. There is a potential selection bias due to the retrospective nature of this study. However, we had a homogeneous cohort of patients with cr-POPF compared with other studies including patients with and without POPF as well as other etiologies of PPH.^26,34,35^ The rates of cr-POPF in our derivation and validation cohorts of patients undergoing pancreatoduodenectomy were comparable with rates previously reported.^2,42,43,44^ Another limitation is that our study is a retrospective analysis of prospectively acquired data and as such does not allow definitive statements regarding changes in management of care for patients. To improve patient outcomes, patients at high risk may benefit from delaying the discharge or intensified postoperative monitoring and other invasive measures (eg, early angiography with prophylactic coiling, and antifungal treatment). However, prospective controlled studies are required to propose recommendations to change the management of care for patients with PPH.

In addition, the isolation of *Candida* species from drain fluid cultures can take up to 3 days, although most PPH occurred after a median of 13 days after surgery. Therefore, the exact timing of cultures remains unclear, but they should be performed as soon as cr-POPF is evident.

## Conclusions

To our knowledge, we developed the first HRS model for the prediction of PPH in the subset of patients with cr-POPF. This model includes easily accessible clinical variables and in this study provided accurate risk stratification in patients diagnosed with cr-POPF. This tool might help clinicians to identify patients at high risk for PPH and initiate early risk mitigation strategies. The model should therefore be validated in further patient cohorts.

## Notes

Supplement 1. **eTable 1.** Clinicopathological Characteristics of Patients in the Derivation and Validation Cohorts **eTable 2.** Univariate Analysis of Predictive Factors for PPH in the Derivation Cohort **eTable 3.** Risk Classification of PPH in the Derivation and Validation Cohorts **eFigure 1.** Distinct Radiological Features **eFigure 2.** Calibration Plots

Supplement 2.
Data Sharing Statement

## References

[1] Wolk S, Grützmann R, Rahbari NN, . Management of clinically relevant postpancreatectomy hemorrhage (PPH) over two decades—a comparative study of 1 450 consecutive patients undergoing pancreatic resection. Pancreatology. 2017;17(6):943-950. doi:10.1016/j.pan.2017.10.006 29111264

[2] Floortje van Oosten A, Smits FJ, van den Heuvel DAF, van Santvoort HC, Molenaar IQ. Diagnosis and management of postpancreatectomy hemorrhage: a systematic review and meta-analysis. HPB (Oxford). 2019;21(8):953-961. doi:10.1016/j.hpb.2019.02.011 30962134

[3] Wente MN, Veit JA, Bassi C, . Postpancreatectomy hemorrhage (PPH): an International Study Group of Pancreatic Surgery (ISGPS) definition. Surgery. 2007;142(1):20-25. doi:10.1016/j.surg.2007.02.001 17629996

[4] Callery MP, Pratt WB, Kent TS, Chaikof EL, Vollmer CM Jr. A prospectively validated clinical risk score accurately predicts pancreatic fistula after pancreatoduodenectomy. J Am Coll Surg. 2013;216(1):1-14. doi:10.1016/j.jamcollsurg.2012.09.002 23122535

[5] Pande R, Halle-Smith JM, Phelan L, ; PARANOIA Study Group; Writing committee; Steering committee. External validation of postoperative pancreatic fistula prediction scores in pancreatoduodenectomy: a systematic review and meta-analysis. [Review]. HPB (Oxford). 2022;24(3):287-298. doi:10.1016/j.hpb.2021.10.006 34810093

[6] McMillan MT, Malleo G, Bassi C, . Multicenter, prospective trial of selective drain management for pancreatoduodenectomy using risk stratification. Ann Surg. 2017;265(6):1209-1218. doi:10.1097/SLA.0000000000001832 27280502

[7] Bassi C, Marchegiani G, Dervenis C, ; International Study Group on Pancreatic Surgery (ISGPS). The 2016 update of the International Study Group (ISGPS) definition and grading of postoperative pancreatic fistula: 11 years after. Surgery. 2017;161(3):584-591. doi:10.1016/j.surg.2016.11.014 28040257

[8] World Medical Association. World Medical Association Declaration of Helsinki: ethical principles for medical research involving human subjects. JAMA. 2013;310(20):2191-2194. doi:10.1001/jama.2013.281053 24141714

[9] Moons KG, Altman DG, Reitsma JB, . Transparent Reporting of a Multivariable Prediction Model for Individual Prognosis or Diagnosis (TRIPOD): explanation and elaboration. Ann Intern Med. 2015;162(1):W1-73. doi:10.7326/M14-0698 25560730

[10] Șandra-Petrescu F, Tzatzarakis E, Mansour Basha M, . Impact of spleen preservation on the incidence of postoperative pancreatic fistula after distal pancreatectomy: is less more? Pancreatology. 2022;22(7):1013-1019. doi:10.1016/j.pan.2022.07.012 35945100

[11] Téoule P, Rasbach E, Oweira H, . Obesity and pancreatic cancer: a matched-pair survival analysis. J Clin Med. 2020;9(11):3526. doi:10.3390/jcm9113526 33142763 PMC7693315

[12] Birgin E, Hablawetz P, Téoule P, Rückert F, Wilhelm TJ. Chronic pancreatitis and resectable synchronous pancreatic carcinoma: a survival analysis. Pancreatology. 2018;18(4):394-398. doi:10.1016/j.pan.2018.04.009 29716797

[13] Tol JA, Gouma DJ, Bassi C, ; International Study Group on Pancreatic Surgery. Definition of a standard lymphadenectomy in surgery for pancreatic ductal adenocarcinoma: a consensus statement by the International Study Group on Pancreatic Surgery (ISGPS). Surgery. 2014;156(3):591-600. doi:10.1016/j.surg.2014.06.016 25061003 PMC7120678

[14] Brodsky JT, Turnbull AD. Arterial hemorrhage after pancreatoduodenectomy. The “sentinel bleed”. Arch Surg. 1991;126(8):1037-1040. doi:10.1001/archsurg.1991.01410320127019 1863209

[15] Nitschke P, Volk A, Welsch T, . Impact of intraoperative re-resection to achieve R0 status on survival in patients with pancreatic cancer: a single-center experience with 483 patients. Ann Surg. 2017;265(6):1219-1225. doi:10.1097/SLA.0000000000001808 27280512

[16] Bassi C, Dervenis C, Butturini G, ; International Study Group on Pancreatic Fistula Definition. Postoperative pancreatic fistula: an international study group (ISGPF) definition. Surgery. 2005;138(1):8-13. doi:10.1016/j.surg.2005.05.001 16003309

[17] Koch M, Garden OJ, Padbury R, . Bile leakage after hepatobiliary and pancreatic surgery: a definition and grading of severity by the International Study Group of Liver Surgery. Surgery. 2011;149(5):680-688. doi:10.1016/j.surg.2010.12.002 21316725

[18] Marchegiani G, Barreto SG, Bannone E, ; International Study Group for Pancreatic Surgery. Postpancreatectomy acute pancreatitis (PPAP): definition and grading from the International Study Group for Pancreatic Surgery (ISGPS). Ann Surg. 2022;275(4):663-672. doi:10.1097/SLA.0000000000005226 34596077

[19] Wente MN, Bassi C, Dervenis C, . Delayed gastric emptying (DGE) after pancreatic surgery: a suggested definition by the International Study Group of Pancreatic Surgery (ISGPS). Surgery. 2007;142(5):761-768. doi:10.1016/j.surg.2007.05.005 17981197

[20] Treckmann J, Paul A, Sotiropoulos GC, . Sentinel bleeding after pancreaticoduodenectomy: a disregarded sign. J Gastrointest Surg. 2008;12(2):313-318. doi:10.1007/s11605-007-0361-2 17952516

[21] Yamauchi FI, Ortega CD, Blasbalg R, Rocha MS, Jukemura J, Cerri GG. Multidetector CT evaluation of the postoperative pancreas. Radiographics. 2012;32(3):743-764. doi:10.1148/rg.323105121 22582357

[22] Raman SP, Horton KM, Cameron JL, Fishman EK. CT after pancreaticoduodenectomy: spectrum of normal findings and complications. AJR Am J Roentgenol. 2013;201(1):2-13. doi:10.2214/AJR.12.9647 23789653

[23] Puppala S, Patel J, McPherson S, Nicholson A, Kessel D. Hemorrhagic complications after Whipple surgery: imaging and radiologic intervention. AJR Am J Roentgenol. 2011;196(1):192-197. doi:10.2214/AJR.10.4727 21178067

[24] Riley RD, Ensor J, Snell KIE, . Calculating the sample size required for developing a clinical prediction model. BMJ. 2020;368:m441. doi:10.1136/bmj.m441 32188600

[25] Steyerberg EW, Harrell FE Jr, Borsboom GJ, Eijkemans MJ, Vergouwe Y, Habbema JD. Internal validation of predictive models: efficiency of some procedures for logistic regression analysis. J Clin Epidemiol. 2001;54(8):774-781. doi:10.1016/S0895-4356(01)00341-9 11470385

[26] Han GJ, Kim S, Lee NK, . Prediction of late postoperative hemorrhage after Whipple procedure using computed tomography performed during early postoperative period. Korean J Radiol. 2018;19(2):284-291. doi:10.3348/kjr.2018.19.2.284 29520186 PMC5840057

[27] Tien YW, Lee PH, Yang CY, Ho MC, Chiu YF. Risk factors of massive bleeding related to pancreatic leak after pancreaticoduodenectomy. J Am Coll Surg. 2005;201(4):554-559. doi:10.1016/j.jamcollsurg.2005.05.007 16183493

[28] Wei HK, Wang SE, Shyr YM, . Risk factors for post-pancreaticoduodenectomy bleeding and finding an innovative approach to treatment. Dig Surg. 2009;26(4):297-305. doi:10.1159/000228245 19602889

[29] Wellner UF, Kulemann B, Lapshyn H, . Postpancreatectomy hemorrhage—incidence, treatment, and risk factors in over 1,000 pancreatic resections. J Gastrointest Surg. 2014;18(3):464-475. doi:10.1007/s11605-013-2437-5 24448997

[30] Izumo W, Higuchi R, Yazawa T, Uemura S, Shiihara M, Yamamoto M. Evaluation of preoperative risk factors for postpancreatectomy hemorrhage. Langenbecks Arch Surg. 2019;404(8):967-974. doi:10.1007/s00423-019-01830-w 31650216 PMC6935390

[31] Uggeri F, Nespoli L, Sandini M, . Analysis of risk factors for hemorrhage and related outcome after pancreatoduodenectomy in an intermediate-volume center. Updates Surg. 2019;71(4):659-667. doi:10.1007/s13304-019-00673-w 31376077

[32] Welsch T, Eisele H, Zschäbitz S, Hinz U, Büchler MW, Wente MN. Critical appraisal of the International Study Group of Pancreatic Surgery (ISGPS) consensus definition of postoperative hemorrhage after pancreatoduodenectomy. Langenbecks Arch Surg. 2011;396(6):783-791. doi:10.1007/s00423-011-0811-x 21611815

[33] Yekebas EF, Wolfram L, Cataldegirmen G, . Postpancreatectomy hemorrhage: diagnosis and treatment; an analysis in 1669 consecutive pancreatic resections. Ann Surg. 2007;246(2):269-280. doi:10.1097/01.sla.0000262953.77735.db 17667506 PMC1933568

[34] Abe K, Kitago M, Shinoda M, . High risk pathogens and risk factors for postoperative pancreatic fistula after pancreatectomy; a retrospective case-controlled study. Int J Surg. 2020;82:136-142. doi:10.1016/j.ijsu.2020.08.035 32861892

[35] Sato A, Masui T, Nakano K, . Abdominal contamination with *Candida albicans* after pancreaticoduodenectomy is related to hemorrhage associated with pancreatic fistulas. Pancreatology. 2017;17(3):484-489. doi:10.1016/j.pan.2017.03.007 28336225

[36] Naglik JR, Challacombe SJ, Hube B. Candida albicans secreted aspartyl proteinases in virulence and pathogenesis. Microbiol Mol Biol Rev. 2003;67(3):400-428. doi:10.1128/MMBR.67.3.400-428.2003 12966142 PMC193873

[37] Vergidis P, Clancy CJ, Shields RK, . Intra-abdominal candidiasis: the importance of early source control and antifungal treatment. PLoS One. 2016;11(4):e0153247. doi:10.1371/journal.pone.0153247 27123857 PMC4849645

[38] Shan YS, Sy ED, Wang ST, Lee JC, Lin PW. Early presumptive therapy with fluconazole for occult *Candida* infection after gastrointestinal surgery. World J Surg. 2006;30(1):119-126. doi:10.1007/s00268-005-7807-z 16369711

[39] Bassetti M, Righi E, Ansaldi F, . A multicenter multinational study of abdominal candidiasis: epidemiology, outcomes and predictors of mortality. Intensive Care Med. 2015;41(9):1601-1610. doi:10.1007/s00134-015-3866-2 26077063

[40] Palumbo D, Tamburrino D, Partelli S, . Before sentinel bleeding: early prediction of postpancreatectomy hemorrhage (PPH) with a CT-based scoring system. Eur Radiol. 2021;31(9):6879-6888. doi:10.1007/s00330-021-07788-y 33665718

[41] Grendar J, Jutric Z, Leal JN, . Validation of fistula risk score calculator in diverse North American HPB practices. HPB (Oxford). 2017;19(6):508-514. doi:10.1016/j.hpb.2017.01.021 28233672

[42] Correa-Gallego C, Brennan MF, D’Angelica MI, . Contemporary experience with postpancreatectomy hemorrhage: results of 1,122 patients resected between 2006 and 2011. J Am Coll Surg. 2012;215(5):616-621. doi:10.1016/j.jamcollsurg.2012.07.010 22921325

[43] Keck T, Wellner UF, Bahra M, . Pancreatogastrostomy Versus Pancreatojejunostomy for Reconstruction After Pancreatoduodenectomy (RECOPANC, DRKS 00000767): perioperative and long-term results of a multicenter randomized controlled trial. Ann Surg. 2016;263(3):440-449. doi:10.1097/SLA.0000000000001240 26135690 PMC4741417

[44] Welsch T, Müssle B, Korn S, . Pancreatoduodenectomy with or without prophylactic falciform ligament wrap around the hepatic artery for prevention of postpancreatectomy haemorrhage: randomized clinical trial (PANDA trial). Br J Surg. 2021;109(1):37-45. 34746958 10.1093/bjs/znab363

