# Supplementary material for: Development and Validation of a Model for Postpancreatectomy Hemorrhage Risk
Source: JAMA Netw Open. 2023 Dec 6;6(12):e2346113. doi: 10.1001/jamanetworkopen.2023.46113 (PMC10701614; doi:10.1001/jamanetworkopen.2023.46113)
Supplement: Supplement 1. — eTable 1. Clinicopathological Characteristics of Patients in the Derivation and Validation Cohorts eTable 2. Univariate Analysis of Predictive Factors for PPH in the Derivation Cohort eTable 3. Risk Classification of PPH in the Derivation and Validation Cohorts eFigure 1. Distinct Radiological Features eFigure 2. Calibration Plots [file jamanetwopen-e2346113-s001.pdf]

Supplementary Online Content

Birgin E, Hempel S, Reeg A, et al. Development and validation of a model for postpancreatectomy hemorrhage risk. *JAMA Netw Open*. 2023;6(12):e2346113. doi:10.1001/jamanetworkopen.2023.46113

eTable 1. Clinicopathological Characteristics of Patients in the Derivation and Validation Cohorts

eTable 2. Univariate Analysis of Predictive Factors for PPH in the Derivation Cohort

eTable 3. Risk Classification of PPH in the Derivation and Validation Cohorts

eFigure 1. Distinct Radiological Features

eFigure 2. Calibration Plots

This supplementary material has been provided by the authors to give readers additional information about their work.

© 2023 Birgin E et al. *JAMA Network Open.*

eTable 1: Clinicopathological characteristics of patients in the derivation and validation cohorts

	Total cohort N = 293	Derivation cohort N = 139	Validation cohort N = 154	P
**Age (years)** [a]	69 (60 – 76)	70 (60 – 77)	68 (59 – 76)	0.224
**BMI (kg/m^2)** [a]	26 (24 – 30)	26 (23 – 29)	27 (24 – 30)	0.260
**Sex ratio (Male:Female)**	187:106	84:55	103:51	0.274
**ASA**				0.132
I	11 (4)	7 (5)	4 (3)	
II	123 (42)	66 (47)	57 (37)	
III	151 (51)	62 (45)	89 (58)	
IV	8 (3)	4 (3)	4 (3)	
**Diagnosis**				0.275
PDAC	71 (24)	38 (27)	33 (21)	
Other	222 (76)	101 (73)	121 (78)	
**Neoadjuvant chemotherapy**	11 (38)	2 (1)	9 (6)	0.064
**Preoperative diabetes**	64 (22)	36 (26)	28 (18)	0.121
**Pulmonary comorbidities**	45 (15)	23 (17)	22 (14)	0.629
**Cardiovascular comorbidities**	92 (31)	64 (46)	28 (18)	< 0.001
**Antithrombotic drugs**	48 (16)	41 (30)	7 (5)	< 0.001
**Blood loss** [a]	500 (300 – 700)	500 (400 – 700)	500 (300 – 600)	0.975
**Venous resection**	37 (13)	16 (12)	21 (14)	0.603
**Surgical revision**	117 (40)	50 (36)	67 (44)	0.192
Completion pancreatectomy	60 (21)	28 (20)	32 (21)	<0.99
**Postoperative complications** [b]				0.189
Grade I - IIIa	156 (53)	80 (58)	76 (49)	
Grade IIIb - IVb	94 (32)	42 (30)	52 (34)	
**90-day mortality rate**	43 (15)	17 (12)	26 (17)	0.322

BMI body mass index, ASA American Society of Anesthesiologists, PDAC pancreatic adenocarcinoma, PPH postpancreatectomy hemorrhage,

[a] Values are median (interquartile range), [b] in line with the Clavien-Dindo classification

© 2023 Birgin E et al. *JAMA Network Open.*

eTable 2: Univariate Analysis of predictive factors for PPH in the derivation cohort

	OR	95%CI	P
**Demographic features**			
Age (years)	1.02	0.98 – 1.06	0.335
BMI (kg/m^2)	1.05	0.96 – 1.15	0.252
Male vs. Female Sex	3.00	0.94 – 9.52	0.062
ASA			
II vs. I	<0.1	0 – NA	0.992
III vs. I	<0.1	0 – NA	0.991
IV vs. I	<0.1	0 – NA	< 0.99
Cardiovascular comorbidities (Yes vs. No)	1.52	0.59 – 3.94	0.387
Pulmonary comorbidities (Yes vs. No)	1.87	0.60 – 5.79	0.277
Chronic pancreatitis (Yes vs. No)	2.23	0.64 – 7.67	0.208
Preoperative Diabetes (Yes vs. No)	0.95	0.32 – 2.82	0.921
Neoadjuvant chemotherapy (Yes vs. No)	6.21	0.37 – 104.00	0.203
Antithrombotic drugs (Yes vs. No)	1.46	0.53 – 4.02	0.466
Diagnosis			
PDAC vs. other	0.42	0.12 – 1.54	0.191
**Preoperative laboratory tests** *			
Albumin (g/l)	1.05	0.96 – 1.16	0.271
Bilirubin (mg/dl)	0.99	0.88 – 1.10	0.787
AP (U/l)	1.00	0.99 – 1.00	0.634
gGT (U/l)	1.00	0.99 – 1.00	0.426
AST (U/l)	1.01	0.99 – 1.01	0.119
ALT (U/l)	1.00	0.99 – 1.01	0.335
Amylase (U/l)	0.99	0.99 – 1.10	0.466
Platelets (x10^9/l)	0.99	0.99 – 1.00	0.208
**Operative features**			
Pylorus-preserving vs. Whipple procedure	0.99	0.20 – 4.180	0.991
Total blood loss	1.00	0.99 – 1.00	0.713
Venous resection	2.23	0.64 – 7.76	0.208
**Postoperative sentinel bleeding**	35.00	10.20 – 120.00	< 0.001
**Postoperative radiologic features**			
Fluid collection with gas	18.70	5.66 – 61.50	< 0.001
Fluid collection with rim enhancement	10.40	3.66 – 29.70	< 0.001
**Postoperative drainage culture**			
Bacteroides spp. (Yes vs. No)	2.82	0.67 – 12.00	0.159
Enterococcus spp. (Yes vs. No)	1.42	0.55 – 3.68	0.468
Escherichia coli (Yes vs. No)	2.57	0.87 – 7.61	0.810
Klebsiella spp. (Yes vs. No)	2.15	0.69 – 6.72	0.190
Prevotella spp. (Yes vs. No)	1.51	0.16 – 14.30	0.718
Pseudomonas spp. (Yes vs. No)	6.21	0.37 – 104.00	0.203
Staphylococcus spp. (Yes vs. No)	0.28	0.03 – 2.23	0.228
Streptococcus spp. (Yes vs. No)	0.99	0.11 – 8.70	0.994
Stenotropha spp. (Yes vs. No)	4.30	0.67 – 27.50	0.124
Candida spp. (Yes vs. No)	7.50	2.65 – 21.20	< 0.001

BMI body mass index, ASA American Society of Anesthesiologists, PDAC pancreatic adenocarcinoma, PPH postpancreatectomy hemorrhage, spp. species
POD postoperative day, OR odds ratio, CI confidence interval, NA not available

© 2023 Birgin E et al. *JAMA Network Open.*

eTable 3: Risk classification of PPH in the derivation and validation cohorts

		Derivation cohort			Validation Cohort		
**Risk category**	**Total Points**	**Patients (N=139)**	**PPH (N=20)**	**PPH rate % (95% CI)**	**Patients (N=154)**	**PPH (N=54)**	**PPH rate % (95% CI)**
**Low**	0 – 1	109	1	1.2 (0 – 6.5)	82	10	12.2 (6.0 – 21.3)
**High**	2 – 5	30	19	63.3 (43.9 – 80.1)	72	44	61.1 (48.9 – 72.4)

PPH postpancreatectomy hemorrhage, CI confidence interval

© 2023 Birgin E et al. *JAMA Network Open.*

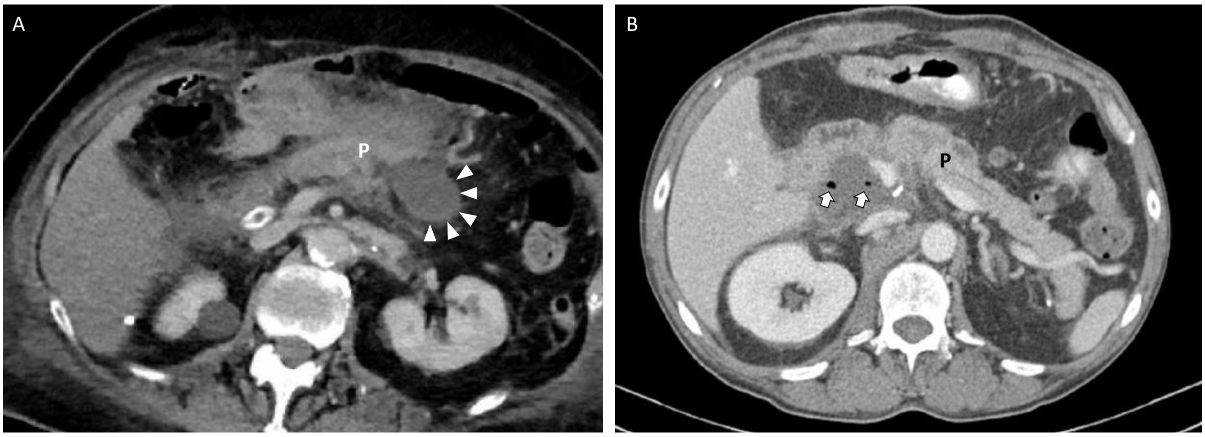

eFigure 1: Distinct radiological features

Fluid collection with rim enhancement (indicated by white triangles, A) and perianastomotic gas (indicated by white arrows, B) is demonstrated in two patients who developed late postpancreatectomy hemorrhage. P pancreatic remnant

© 2023 Birgin E et al. *JAMA Network Open.*

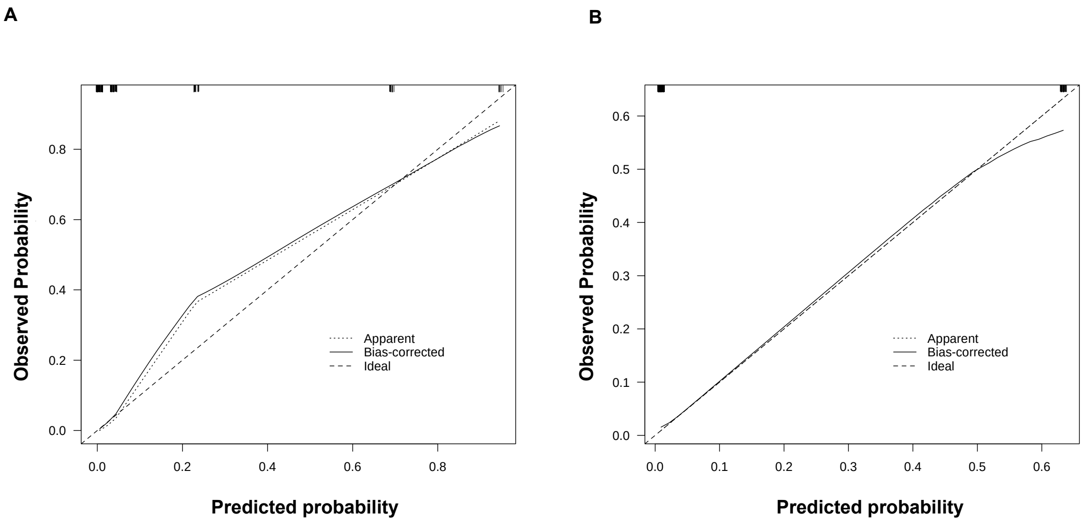

eFigure 2: Calibration plots

Calibration plots of observed against predicted values of postpancreatectomy hemorrhage in the derivation cohort with regard to the hemorrhage risk score model using "0-5 points" (A) and the risk category model using "0-1, ≥ 2 points" (B). The apparent model and bias-corrected (overfitting-corrected) bootstrap model plot is displayed. A calibration slope of 1 would indicate a perfect calibration model.

© 2023 Birgin E et al. *JAMA Network Open.*
