# Supplementary material for: Development and Validation of a Model for Postpancreatectomy Hemorrhage Risk
Source: JAMA Netw Open. 2023 Dec 6;6(12):e2346113. doi: 10.1001/jamanetworkopen.2023.46113 (PMC10701614; doi:10.1001/jamanetworkopen.2023.46113)
Supplement: Supplement 2. — Data Sharing Statement [file jamanetwopen-e2346113-s002.pdf]

Data Sharing Statement

Birgin. Development and Validation of a Model for Postpancreatectomy Hemorrhage Risk. *JAMA Netw Open*. Published December 06, 2023. doi:10.1001/jamanetworkopen.2023.46113

Data
Data available: No

Additional Information
Explanation for why data not available: The data that support the findings of this study are available from the corresponding author upon reasonable request
